# Creep damage model of deep granite under coupled temperature-stress conditions

**DOI:** 10.1038/s41598-026-44291-0

**Published:** 2026-03-18

**Authors:** Jun Hu, Junxin Shi, Jin Wu, Shuai Zhang, Yamin Zhao, Shangjun Zhao, Zhaokui Sun, Hanyu Dang, Houhai Yuan

**Affiliations:** 1 School of Civil and Architectural Engineering, Hainan University, Haikou, 570228 Hainan China; 2 Marine Science and Technology Collaborative Innovation Center, Hainan University, Haikou, 570228 Hainan China; 3 State Key Laboratory of Tropic Ocean Engineering Materials and Materials Evaluation, Hainan University, Haikou, 570228 Hainan China; 4 School of Information and Communication Engineering, Hainan University, Haikou, 570228 Hainan China; 5 Shaanxi Zhuoxin Engineering Testing Co., Ltd., Xian, 710000 Shanxi China; 6 Hainan Nonferrous Engineering Survey and Design Institute Co., Ltd. Haikou, Hainan, 570228 China

**Keywords:** Temperature–stress coupling, Damage factor, Yield criterion, Creep model, Parameter degradation, Engineering, Natural hazards, Solid Earth sciences

## Abstract

To investigate the long-term creep behavior of deep granite in geological repositories for high-level radioactive waste under coupled temperature and stress conditions, a full-time temperature–stress coupled creep damage constitutive model (TSD model) was developed. The model incorporates a temperature-induced damage factor ${D}_{T}$, a stress-induced damage factor ${D}_{S}$, and a coupled damage factor ${D}_{TS}$. Based on the classical Nishihara model, the elastic, viscous, and viscoplastic components are modified to better capture creep behavior under complex thermo–mechanical conditions. An improved Drucker–Prager yield criterion is introduced to account for temperature and stress effects on cohesion and internal friction angle, enabling accurate description under high temperature and triaxial stress. Triaxial creep test data of granite at 23 °C, 50 °C, and 90 °C are used for validation. Model parameters are identified through a hybrid optimization strategy combining global search and local least-squares fitting. The TSD model successfully reproduces the three typical creep stages of granite, achieving excellent agreement with experiments (R² > 0.99), especially in the nonlinear accelerating phase. Results show that increasing temperature significantly accelerates mechanical degradation, reduces cohesion and internal friction angle, and leads to loss of shear strength. This study provides a theoretical basis for evaluating the long-term stability of host rocks in deep geological disposal of high-level radioactive waste and offers a framework for extending the model to broader temperature ranges and complex environments such as seepage and chemical coupling.

## Introduction

In deep geological disposal projects, the repository often encounters issues such as excessive deformation of the surrounding rock, loss of effective volume, and even failure of storage functionality during both the construction and operational phases. These problems arise not only from internal pressure fluctuations and roof collapse but are also closely related to the creep behavior of the host rock itself^1–3^. Unlike conventional deep underground structures, high-level radioactive waste (HLW) repositories are subjected to continuous heat release from radionuclide decay, which significantly raises the temperature of the surrounding rock. This thermal effect alters the rock’s mechanical properties, promotes the initiation and propagation of fractures, and potentially creates pathways for radionuclide migration^4,5^. Therefore, investigating the long-term evolution of rock creep behavior under high-temperature conditions and developing appropriate constitutive models are essential for evaluating the long-term safety of HLW geological repositories.

Granite, due to its high stability, strength, and low permeability, has been regarded as an ideal host rock for HLW disposal, and is one of the primary candidate rock types in disposal projects worldwide^6^. To date, a large number of uniaxial and triaxial creep experiments have been conducted on granite specimens under various temperatures and loading paths. These studies have focused on the damage evolution during the creep process and have led to the development of numerous constitutive models capable of capturing the creep characteristics of granite. Currently, two main approaches are adopted in constructing creep models: (1) Empirical creep equations derived from extensive experimental data fitting^7–9^; (2) Modification of classical creep models by introducing nonlinear components to replace linear elements, thereby capturing the nonlinear behavior throughout the full creep process^10–12^. However, approach (1) heavily depends on large volumes of test data, limiting its applicability. Its simplified mathematical form also fails to represent the actual rock behavior under complex stress conditions, and as a result, it has gradually fallen out of favor among researchers. Although approach (2) offers improved predictive capability, it may still produce considerable errors under complex conditions such as high temperature and elevated geostress.

To overcome the limitations of the aforementioned methods, researchers have incorporated damage mechanics theory into approach (2), introducing damage variables into the fundamental constitutive relationships to enhance model performance. For example, Zhao et al.^13^ suggested that damage in rocks is relatively minor during the primary and steady-state creep stages, and thus introduced a nonlinear viscoplastic element accounting for damage only in the accelerating stage, establishing a complete viscoelastoplastic creep damage model. Zhang et al.^14,15^, based on nonlinear rheological theory and damage mechanics, developed a new creep model by serially connecting an elastic element, a nonlinear Kelvin body, a viscous element, and a damage-based viscoplastic component. Lyu et al.^16^ proposed a nonlinear damage model capable of representing the long-term brittle creep behavior of granite by combining uniaxial creep experiments with fractional derivative theory. Sun et al.^17^, considering the deep tunnel environment, developed a model coupling strain softening and creep damage, which was successfully implemented in FLAC3D for numerical simulations. Cheng et al.^18^, based on the Weibull damage law, coupled a viscoelastic body and a nonlinear viscoplastic body with the classical Western model to establish a model that can better describe the accelerated creep behavior of soft and hard rocks. In addition, several researchers have considered the influence of high temperature on rock creep behavior and introduced thermodynamic principles to construct temperature-dependent damage models. For instance, Xu et al.^19^ conducted high-temperature gradient loading creep tests and scanning electron microscopy (SEM) observations to systematically analyze creep deformation and mechanical degradation mechanisms under thermo-mechanical coupling. Wang et al.^20^ established a statistical damage model based on high-temperature dynamic creep tests and Weibull distribution theory, capable of describing the effects of temperature and strain rate. Liu et al.^21^ proposed a temperature damage creep model by incorporating the Caputo function associated with strain relaxation and validated it using sandstone experiments under multiple temperature conditions. Pan et al.^22^, based on high-temperature creep tests of red sandstone, introduced the Harris function to modify the viscous component, leading to the development of a Bingham-type model that accurately captures the accelerating creep stage under elevated temperatures. Jiang et al.^23^ developed an improved Burgers temperature damage model through triaxial creep experiments involving thermal cycling of quartz sandstone, and further verified the model through meso-scale experimental observations. Liang et al.^24^ introduced temperature and mechanical damage variables into the viscoelastic element to establish a new three-dimensional creep constitutive model, and conducted a sensitivity analysis of the parameters.

Despite the significant progress achieved in the aforementioned studies, two key issues remain to be addressed. First, under three-dimensional stress conditions, the selection of creep yield criteria is often inadequate. Most existing models substitute the stress or long-term strength derived from one-dimensional conditions, thereby neglecting the nonlinear effects that dominate during the accelerating creep stage. Additionally, commonly adopted criteria such as the Mises and Tresca yield functions, while simplifying the modeling process, deviate from actual rock behavior. Second, the physical interpretation of damage factors remains ambiguous and lacks a consistent quantitative definition. In some models, both the elastic modulus and viscous coefficient are treated as a one-dimensional function of a damage variable. However, given that the viscous coefficient represents a time derivative associated with creep rate, such treatment introduces theoretical inconsistencies^25,26^.

To address these limitations, a thermo–stress coupled creep damage model (TSD model) with full-timescale nonlinear characteristics is proposed in this study. Unlike conventional Nishihara-type extensions that merely introduce empirical damage coefficients to modify material parameters, the proposed model establishes a thermo–mechanical damage evolution framework in which thermal degradation, stress-induced damage accumulation, and their coupled interaction are explicitly distinguished. First, a coupled damage factor is formulated to describe the progressive degradation of mechanical properties under combined temperature and stress conditions, and the constitutive relationships of the elastic and viscous components are revised accordingly. Second, in the plastic stage, the Drucker–Prager yield criterion is improved by incorporating hydrostatic pressure and an intermediate principal stress coefficient, while cohesion and internal friction angle are allowed to evolve dynamically with damage. This enables continuous degradation of the yield surface under thermo–mechanical coupling, rather than a simple parameter reduction. Furthermore, the mathematical expressions of the model are derived under both one-dimensional and three-dimensional stress states. A hybrid parameter identification strategy combining global optimization and local least-squares methods is employed to calibrate the model parameters and analyze the evolution of damage factors during creep. The proposed TSD model provides improved accuracy in characterizing long-term creep behavior of granite under coupled thermo–mechanical conditions and offers theoretical support for stability assessment in deep geological disposal projects.

## Thermo–stress coupled creep damage constitutive model

Existing thermal creep constitutive models often fail to capture the behavior of rocks during the accelerating creep stage. This limitation primarily arises from deficiencies in model construction and the inability of key parameters to reflect their actual evolution during creep. Classical creep constitutive models for rocks are typically composed of fundamental mechanical elements—such as Hookean elastic bodies and Newtonian viscous bodies—arranged in series or parallel to represent different stress–strain relationships^27,28^. In these classical models, parameters such as the elastic modulus and viscosity coefficient are usually treated as constants or assigned a limited set of values. Although the fitted creep curves may align well with experimental data, the resulting parameters often do not capture their true variation during the creep process. As a result, such models are unable to accurately describe the actual evolution of rock deformation over time. Therefore, a fundamental challenge in developing creep constitutive models for rocks under high-temperature conditions lies in how to realistically characterize the evolution of key parameters—such as the elastic modulus and viscosity coefficient—throughout the entire creep process.

### Temperature damage factor

Most rocks are composed of multiple constituents, exhibiting characteristics of polycrystalline composite media and heterogeneity. Under thermo–mechanical coupling, over time (considering time effects), dislocations occur within the rock’s grains, and the combined effects of thermal cracking due to temperature and damage-induced fractures due to stress lead to complex fracture mechanisms. This results in the degradation of the rock’s mechanical properties until creep failure occurs. Therefore, the influence of temperature on the rock’s mechanical performance cannot be ignored^29^. As the temperature increases, the rock’s mechanical properties gradually deteriorate. This deterioration manifests as damage to the mechanical elements, specifically the reduction in elastic modulus and viscosity coefficient^30^. It is assumed that the formation and development of cracks in the rock under thermo–mechanical coupling follow random and Weibull statistical patterns. If macroscopic damage is continuous, the temperature damage factor^31–33^ can be expressed as follows.

1 $$\begin{array}{c}{D}_{T}=1-{exp}\left[-{\left(\frac{{\epsilon}_{1}}{\lambda}\right)}^{m}\right]\end{array}$$

where ${\epsilon}_{1}$ is the axial strain of the rock microelement; *m* (*m* > 0) and *λ* (*λ* > 0) are the Weibull shape and scale parameters, respectively. In this study, the scale parameter λ is normalized to unity without loss of generality, so that the damage evolution is governed primarily by the shape parameter m, thereby avoiding strong parameter correlation during model formulation.

Based on the generalized Hooke’s law and thermodynamic theory^34,35^, the axial strain ${\epsilon}_{1}$ of a microelement under equal confining pressure conditions can be expressed as:

2 $$\begin{array}{c}{\epsilon}_{1}=\frac{1}{E}\left({\sigma}_{1}-2\mu{\sigma}_{3}\right)+\alpha\left(T-{T}_{0}\right)\end{array}$$

where *E* and *ν* are the elastic modulus and Poisson’s ratio, respectively; ${\sigma}_{1}$ and ${\sigma}_{3}$ represent the axial and radial stresses; $T$ is the current temperature, and ${T}_{0}$ denotes the room temperature, taken as 23 °C.

By substituting Eq. (2) into Eq. (1), the following expression is obtained:

3 $$\begin{array}{c}{D}_{T}=1-{exp}\left\{-{\left[\frac{1}{E}\left({\sigma}_{1}-2\mu{\sigma}_{3}\right)+\alpha\left(T-23\right)\right]}^{m}\right\}\end{array}$$

In deep geological formations, rocks are subjected to elevated temperatures, which, at the microscale, primarily influence the connectivity of intergranular fracture networks. Studies^36,37^ have shown that under high-temperature conditions, the fractal dimension of the fracture network connectivity in rocks exhibits a quadratic relationship with temperature. Therefore, it is assumed here that the Weibull shape parameter mmm is associated with the connectivity of the internal fracture network. The relationship between *m* and temperature *T* can thus be expressed as:

4 $$\begin{array}{c}m=a{\left(T-23\right)}^{2}+b\left(T-23\right)+c\end{array}$$

where *a*, *b*, and *c* are fitting coefficients that can be determined through experimental data regression. By substituting Eq. (4) into Eq. (3), the temperature damage factor can be expressed as:

5 $$\begin{array}{c}{D}_{T}=1-{exp}\left\{-{\left[\frac{1}{E}\left({\sigma}_{1}-2\mu{\sigma}_{3}\right)+\alpha\left(T-23\right)\right]}^{a{\left(T-23\right)}^{2}+b\left(T-23\right)+c}\right\}\end{array}$$

The damage factor ${D}_{T}$ is equal to zero at room temperature (${T}_{0}=23\mathrm{℃}$), indicating that no thermal damage occurs in the rock material under ambient conditions. However, the previous equation does not satisfy this boundary condition. Therefore, a temperature-dependent multiplier $f\left(T\right)$ is introduced to the stress term $\frac{1}{E}\left({\sigma}_{1}-2\mu{\sigma}_{3}\right)$ in Eq. (5), as shown in the following expression:

6 $$\begin{array}{c}{D}_{T}=1-{exp}\left\{-{\left[\frac{1}{E}\left({\sigma}_{1}-2\mu{\sigma}_{3}\right)f\left(T\right)+\alpha\left(T-23\right)\right]}^{a{\left(T-23\right)}^{2}+b\left(T-23\right)+c}\right\}\end{array}$$

where $f\left(T\right)$ is a temperature switch function, defined as:

7 $$\begin{array}{c}f\left(T\right)={max}\left(0,T-23\right)\end{array}$$

The temperature switch function $f\left(T\right)$ is introduced to ensure that the thermal damage factor satisfies the boundary condition ${D}_{T}=0$ at room temperature (23 °C). Since the present model focuses on thermo–mechanical behavior under elevated temperature conditions (*T* ≥ room temperature), room temperature is taken as the reference undamaged state. The switch function therefore serves as a normalization constraint rather than an empirical fitting function.

**Fig. 1:**
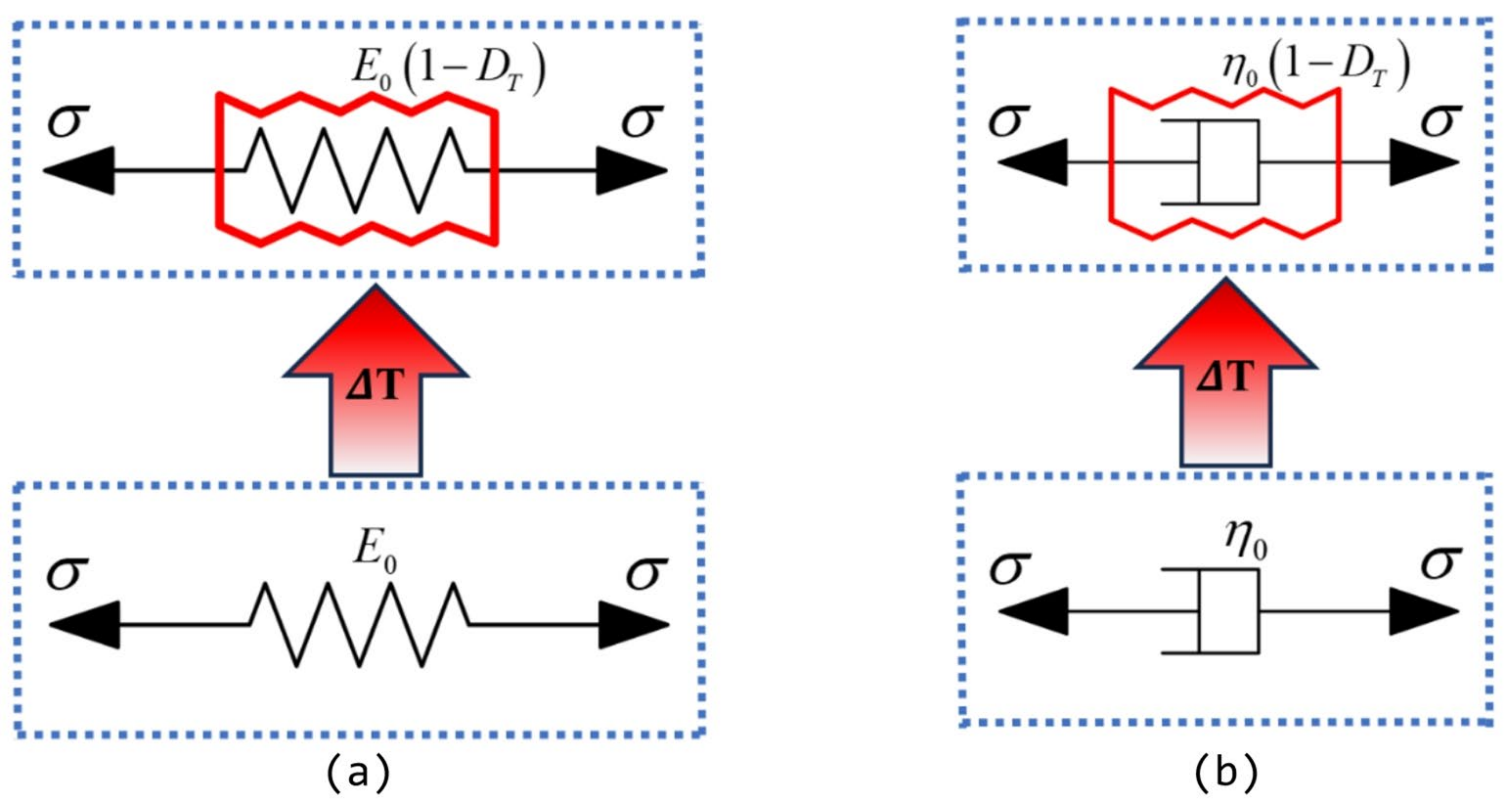
Temperature damage components. (**a**) Temperature damaged elastic element, (**b**) temperature damaged viscous element.

Accordingly, the constitutive relationships of the elastic and viscous components considering thermal damage (as illustrated in Fig. 1) can be expressed by Eqs. (8) and (9), respectively:

8 $$\begin{array}{c}\sigma={E}_{0}\left(1-{D}_{T}\right)\epsilon\end{array}$$

9 $$\begin{array}{c}\sigma={\eta}_{0}\left(1-{D}_{T}\right)\dot{\epsilon}\end{array}$$

where ${E}_{0}$ and ${\eta}_{0}$ are the elastic modulus and viscosity coefficient at room temperature (23 °C), respectively; $0\le{D}_{T}<1$; $\sigma$ is the stress acting on the element; $\epsilon$ and $\dot{\epsilon}$ represent the strain and strain rate of the element, respectively. The thermal damage factor therefore reflects the temperature-induced evolution of fracture network connectivity at the microscale, leading to progressive stiffness degradation of the rock matrix.

Although confining pressure may influence the instantaneous elastic modulus of rocks, in the present formulation the elastic element is assumed to be primarily affected by temperature. This assumption is adopted because the elastic element represents the instantaneous mechanical response prior to significant damage evolution, while the primary objective of the model is to characterize time-dependent creep deformation and strength degradation under thermo–mechanical coupling. The effect of confining pressure on stiffness is implicitly reflected in the experimental initial elastic response used for parameter calibration. Introducing explicit stress-dependent elastic parameters would considerably increase model complexity and parameter coupling, which is beyond the scope of the current study.

### Stress damage factor

Considering temperature effects alone is insufficient to fully capture the actual evolution of certain parameters during the creep process. It is also necessary to account for the influence of stress-induced damage on parameter variation. Studies have shown that internal damage in rocks begins to develop only when the externally applied load reaches or exceeds a certain stress threshold—that is, when the rock enters the yield stage^38^. Once yielding occurs, the rock transitions into the accelerating creep stage. During this stage, the evolution of the damage variable can be described using appropriate mathematical models, and its evolution equation has been defined in related studies^39^.

10 $$\begin{array}{c}\frac{d{D}_{S}}{dt}=C{\left(\frac{\sigma}{1-{D}_{S}}\right)}^{N}\end{array}$$

where *C* and *N* are material-related parameters, and ${D}_{S}$ is the stress-induced damage variable.

By integrating Eq. (10) and applying the boundary conditions $t={t}_{F}$ and *D*=1, the creep failure time of the rock can be obtained as:

11 $$\begin{array}{c}{t}_{F}=\frac{1}{C\left(1+N\right){\sigma}^{N}}\end{array}$$

where ${t}_{F}$ is the time at which creep failure of the rock occurs.

By combining Eqs. (10) and (11), the evolution equation of the stress-induced damage variable with respect to creep time during the creep process can be obtained as:

12 $$\begin{array}{c}{D}_{S}=1-{\left(1-\frac{t}{{t}_{F}}\right)}^{\frac{1}{1+N}}\end{array}$$

**Fig. 2:**
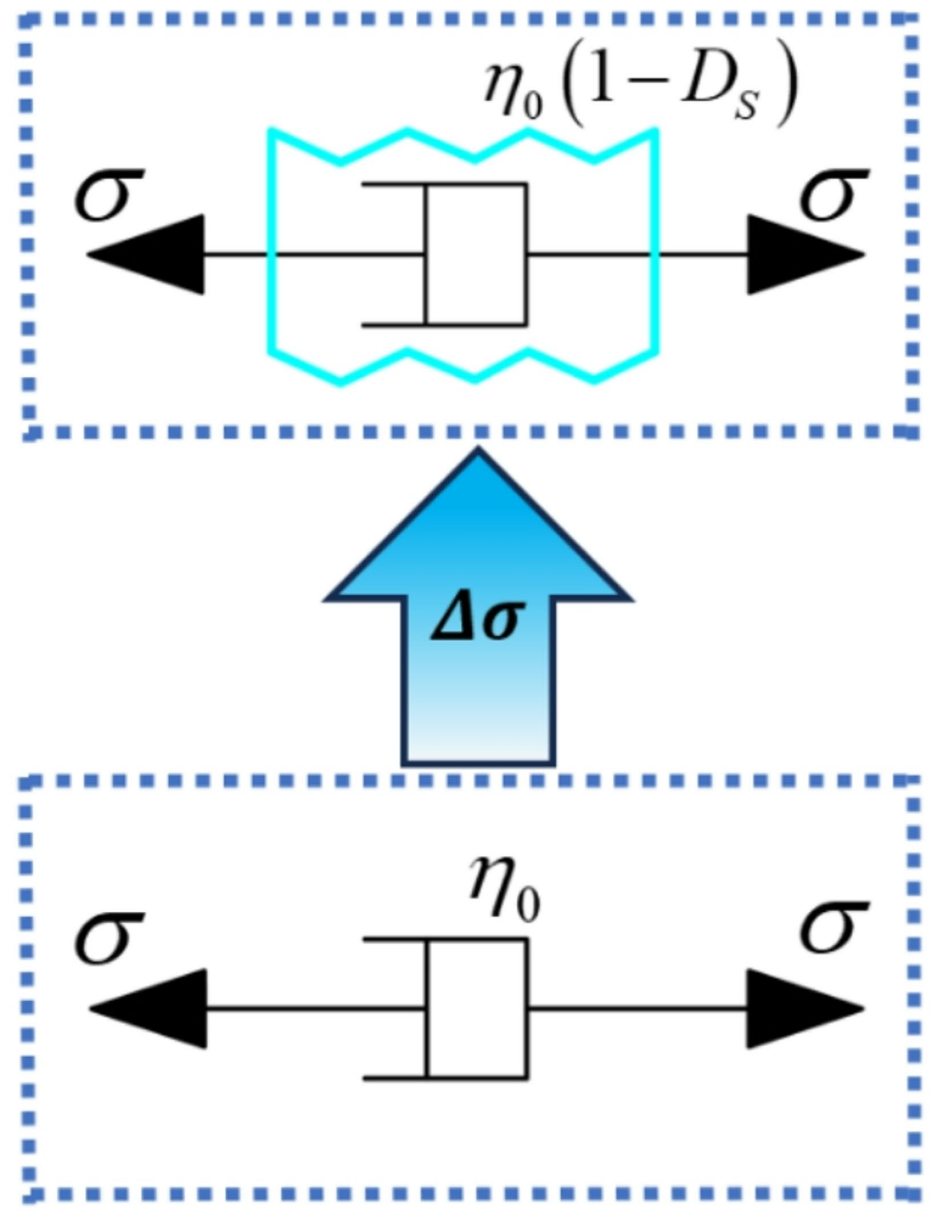
Stress-damaged viscous element.

The stress damage factor characterizes progressive microcrack accumulation after yielding and governs the time-dependent deterioration during the accelerating creep stage. By introducing the stress-induced damage variable ${D}_{S}$ into the viscous component, the constitutive relationship of the viscous element considering stress damage (as shown in Fig. 2) can be obtained. Its constitutive equation is given by Eq. (13):

13 $$\begin{array}{c}\sigma={\eta}_{0}\left(1-{D}_{S}\right)\dot{\epsilon}\end{array}$$

The coupled damage factor under combined temperature and stress conditions is denoted as ${D}_{TS}$, and is defined as follows:

14 $$\begin{array}{c}{D}_{TS}={D}_{T}+{D}_{S}-{D}_{T}{D}_{S}\end{array}$$

That is,

15 $$\begin{array}{c}{D}_{TS}=1-{exp}\left\{-{\left[\frac{1}{E}\left({\sigma}_{1}-2\mu{\sigma}_{3}\right)f\left(T\right)+\alpha\left(T-{T}_{0}\right)\right]}^{a{\left(T-23\right)}^{2}+b\left(T-23\right)+c}\right\}{\left(1-\frac{t}{{t}_{F}}\right)}^{\frac{1}{1+N}}\end{array}$$

where $$0\le{D}_{TS}<1$$.

**Fig. 3:**
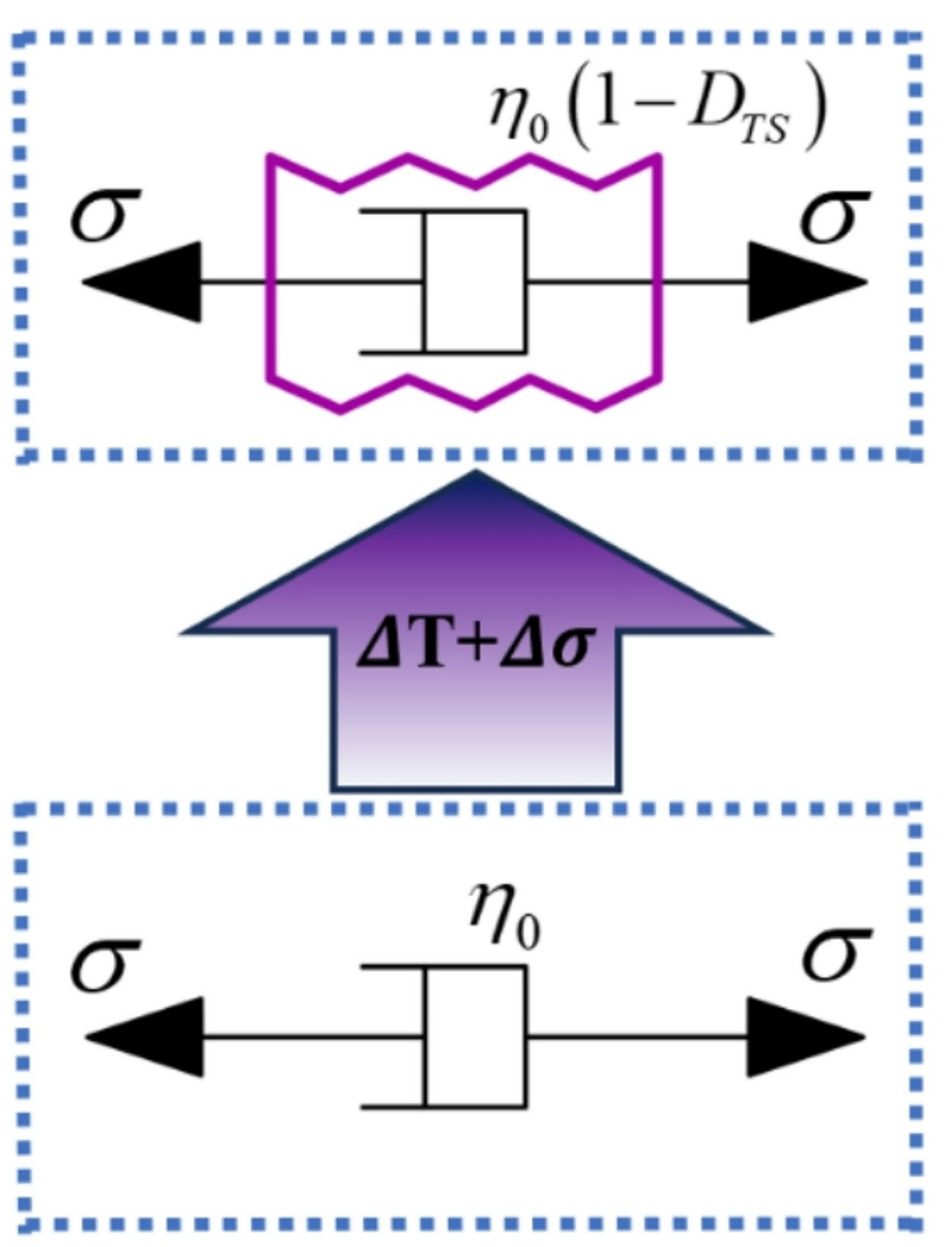
Temperature–stress coupled damaged viscous element.

The coupled damage factor reflects the synergistic interaction between thermal degradation and stress-induced damage during creep. At elevated temperatures, the connectivity of the internal fracture network increases, reducing the material stiffness and facilitating crack initiation. Once the rock enters the yielding stage, stress damage accumulates progressively with time. Under such conditions, thermal weakening and stress-driven crack propagation act simultaneously, leading to accelerated instability during the tertiary creep stage. Therefore, the coupled damage factor governs not only parameter degradation but also the cooperative deterioration mechanism that controls the transition from steady-state creep to accelerating failure. The constitutive relationship of the viscous element considering thermo–stress coupled damage (as illustrated in Fig. 3) is given by:

17 $$\begin{array}{c}\sigma={\eta}_{0}\left(1-{D}_{TS}\right)\dot{\epsilon}\end{array}$$

### Development of the thermo–stress coupled damage (TSD) creep constitutive model

**Fig. 4:**
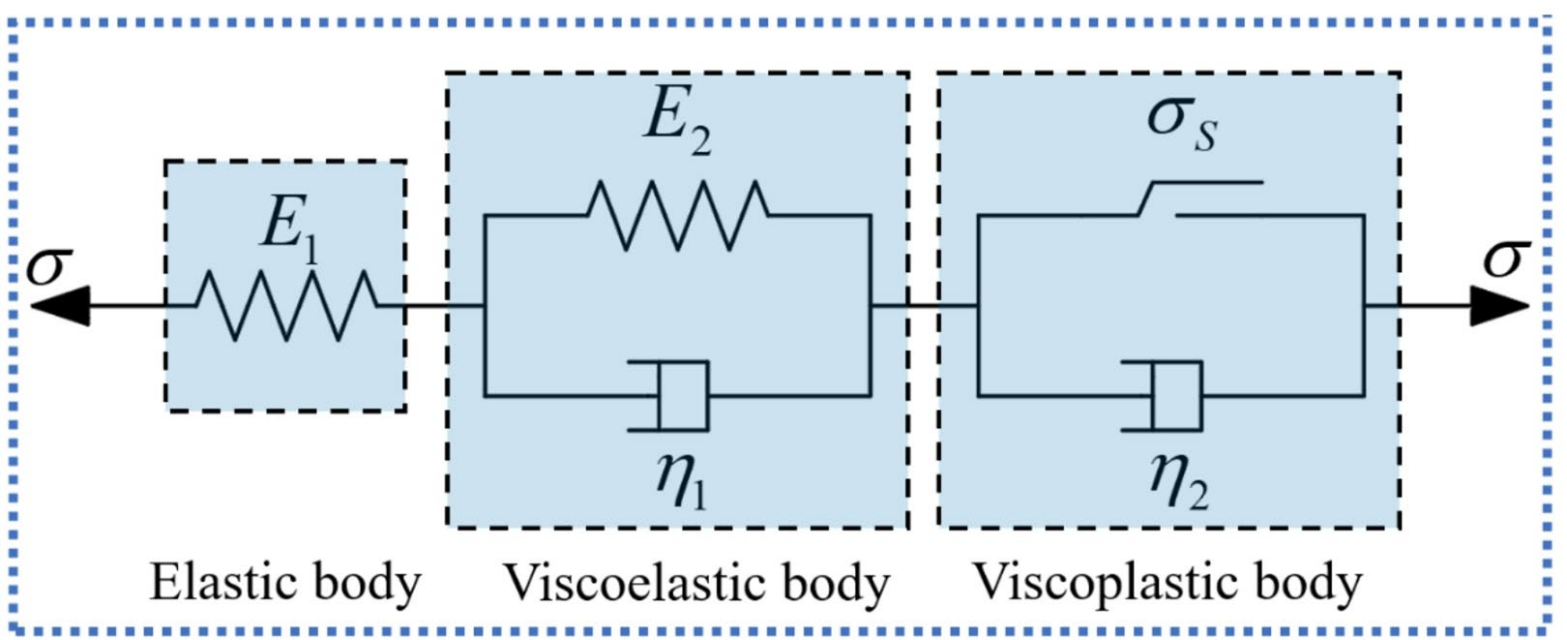
Classical Nishihara model.

The classical Nishihara model, composed of an elastic element, a viscoelastic element, and a viscoplastic element connected in series, has been widely adopted due to its capability to represent the elastic, viscoelastic, and viscoplastic deformation characteristics of rocks. As illustrated in Fig. 4, its one-dimensional creep constitutive equation is given by Eq. (17). However, as engineering environments become increasingly complex, the limitations of the classical Nishihara framework have become more evident. In particular, it is unable to adequately capture the nonlinear accelerating creep stage, does not account for the progressive evolution of mechanical parameters during damage accumulation, and lacks a mechanism to describe yield surface degradation under high-temperature conditions. Although several modified Nishihara-type models introduce damage variables to adjust elastic or viscous coefficients, such approaches generally remain at the level of parameter degradation and do not explicitly describe the thermo–mechanical coupling mechanism governing strength evolution. Therefore, building upon the classical Nishihara model, this study develops a thermo–stress coupled creep damage framework in which stiffness degradation, damage accumulation, and yield surface evolution are systematically incorporated to characterize high-temperature creep behavior of granite.

17 $$\begin{array}{c}\left\{\begin{array}{c}\epsilon=\frac{\sigma}{{E}_{1}}+\frac{\sigma}{{E}_{2}}\left[1-{exp}\left(-\frac{{E}_{2}}{{\eta}_{1}}t\right)\right],\hspace{0.33em}\hspace{0.33em}\sigma<{\sigma}_{s},\\\epsilon=\frac{\sigma}{{E}_{1}}+\frac{\sigma}{{E}_{2}}\left[1-{exp}\left(-\frac{{E}_{2}}{{\eta}_{1}}t\right)\right]+\frac{\sigma-{\sigma}_{s}}{{\eta}_{2}}t,\hspace{0.33em}\hspace{0.33em}\sigma\ge{\sigma}_{s}\end{array}\right.\end{array}$$

where ${E}_{1}$ and ${E}_{2}$ are the elastic moduli of the elastic element and the viscoelastic Kelvin element, respectively; ${\eta}_{1}$ and ${\eta}_{2}$ are the viscosity coefficients of the viscoelastic Kelvin element and the viscoplastic Bingham element, respectively; ${\sigma}_{s}$ is the yield stress of the rock; $\sigma$ and $\epsilon$ represent the applied stress and the strain of the model, respectively; and *t* is the creep time.

**Fig. 5:**
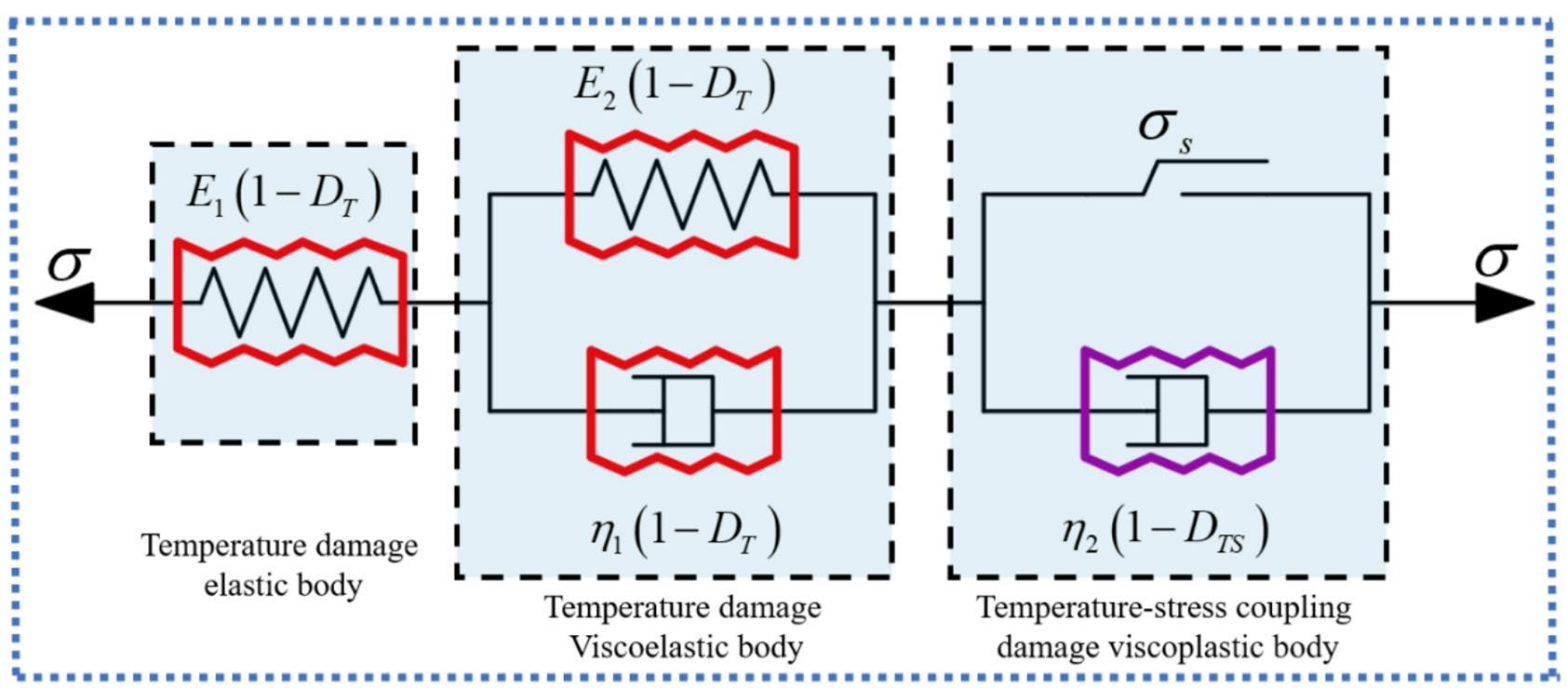
TSD creep constitutive model.

**Fig. 6:**
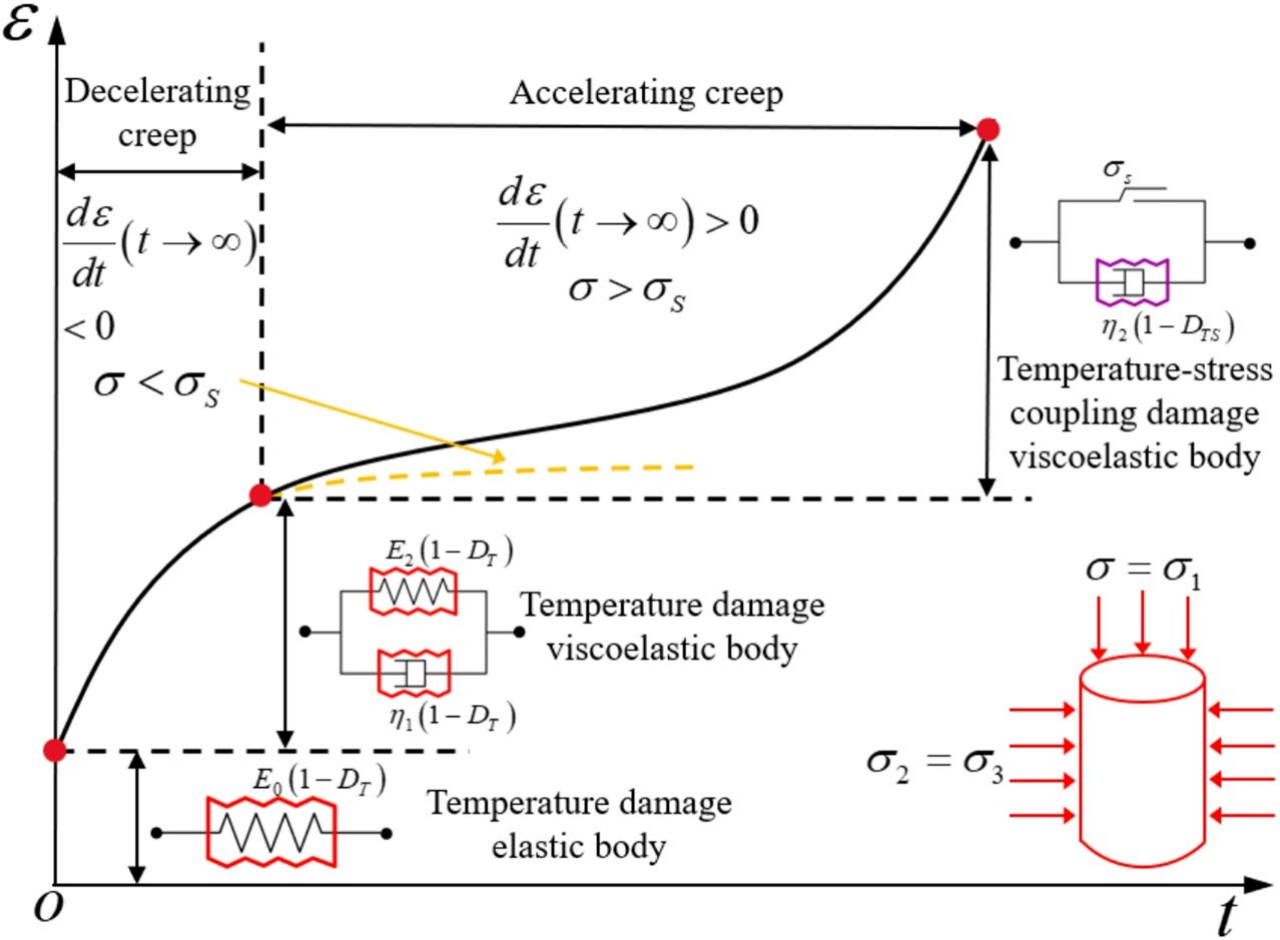
Relationship between the TSD creep constitutive model and the various stages of creep.

In this study, the classical Nishihara model components are replaced by the temperature damaged elastic element, temperature damaged viscous element, and temperature–stress coupled damaged viscous element developed in “Temperature damage factor” and “Stress damage factor”. By comprehensively accounting for the influence of damage effects on model parameters, a thermo–stress coupled damage (TSD) creep constitutive model is established, as illustrated in Fig. 5. The viscoelastic element considering temperature damage is used to characterize the decelerating creep stage, while the viscoplastic element considering temperature-stress coupled damage is used to characterize the accelerating creep stage, as shown in Fig. 6.

By combining Eqs. (8), (9), (16), and (17), the one-dimensional creep constitutive equation of the TSD model can be obtained, as given in Eq. (18):

18 $$\begin{array}{c}\epsilon=\left\{\begin{array}{c}\frac{\sigma}{{E}_{1}\left(1-{D}_{T}\right)}+\frac{\sigma}{{E}_{2}\left(1-{D}_{T}\right)}\left\{1-{exp}\left[-\frac{{E}_{2}\left(1-{D}_{T}\right)}{{\eta}_{1}\left(1-{D}_{T}\right)}t\right]\right\}\text{}\text{}\text{}\sigma\le{\sigma}_{s}\\\frac{\sigma}{{E}_{1}\left(1-{D}_{T}\right)}+\frac{\sigma}{{E}_{2}\left(1-{D}_{T}\right)}\left\{1-{exp}\left[-\frac{{E}_{2}\left(1-{D}_{T}\right)}{{\eta}_{1}\left(1-{D}_{T}\right)}t\right]\right\}\\+\frac{\sigma-{\sigma}_{s}}{{\eta}_{2}\left(1-{D}_{TS}\right)}\text{}\text{}\text{}\text{}\text{}\text{}\text{}\text{}\text{}\text{}\text{}\text{}\text{}\text{}\text{}\text{}\text{}\text{}\text{}\text{}\text{}\text{}\text{}\text{}\text{}\text{}\text{}\text{}\text{}\text{}\text{}\text{}\text{}\text{}\text{}\text{}\text{}\text{}\text{}\text{}\text{}\text{}\text{}\text{}\text{}\text{}\text{}\text{}\text{}\text{}\text{}\text{}\text{}\text{}\text{}\text{}\text{}\text{}\text{}\text{}\text{}\text{}\text{}\text{}\text{}\text{}\text{}\text{}\text{}\text{}\text{}\text{}\text{}\text{}\text{}\text{}\sigma>{\sigma}_{s}\end{array}\right.\end{array}$$

### Three-dimensional form of the TSD creep constitutive model

During the operational period of deep granite repositories, the surrounding rock is continuously subjected to both elevated temperatures and triaxial stress conditions. Therefore, it is necessary to extend the one-dimensional creep damage model to three dimensions in order to analyze the creep damage behavior of deep granite under realistic engineering conditions. Before deriving the three-dimensional constitutive relationship of the TSD creep model, the following assumptions are made^40,41^: (1) Deep granite is considered an isotropic material; (2) Poisson’s ratio remains constant during the creep process. Although Poisson’s ratio may evolve with progressive damage during the tertiary creep stage, it is treated as a constant in the present formulation as a first-order approximation commonly adopted in rock creep modeling. The primary focus of the proposed model is on the deviatoric stress–controlled creep behavior and the evolution of shear strength parameters. Introducing a variable Poisson’s ratio would significantly increase parameter coupling and model complexity, which is beyond the scope of the current study. Future work may incorporate volumetric damage evolution and variable elastic parameters to further improve the description of coupled deformation mechanisms.

It is generally accepted that the deformation of granite is mainly influenced by the deviatoric stress tensor ${S}_{ij}$, while the spherical stress tensor ${\delta}_{ij}{\sigma}_{m}$, which only affects volumetric changes, can be neglected. Based on this assumption, the stress–strain relationship of granite under three-dimensional stress conditions can be expressed from Eq. (18) as:

19 $$\begin{array}{c}\left\{\begin{array}{c}{S}_{ij}={S}_{ij}^{1}={S}_{ij}^{2}={S}_{ij}^{3}\\{\epsilon}_{ij}={\epsilon}_{ij}^{1}+{\epsilon}_{ij}^{2}+{\epsilon}_{ij}^{3}\end{array}\right.\end{array}$$

where ${S}_{ij}$ and ${\epsilon}_{ij}$ represent the deviatoric stress and strain tensors, respectively, and the superscripts 1 to 3 correspond to the different components of the TSD creep constitutive model.

For the strain ${\epsilon}_{ij}^{1}$ of the elastic element considering thermal damage, the expression can be derived based on the generalized Hooke’s law and tensor theory as follows:

20 $$\begin{array}{c}{\epsilon}_{ij}^{1}=\frac{{S}_{ij}^{1}}{2{G}_{1}\left(1-{D}_{T}\right)}+\frac{1}{3K\left(1-{D}_{T}\right)}{\sigma}_{m}{\delta}_{ij}\end{array}$$

where ${\sigma}_{m}{\delta}_{ij}$ is the spherical (hydrostatic) stress tensor, *K* is the bulk modulus of the rock, and ${G}_{1}$ is the shear modulus of the elastic element considering thermal damage.

For the strain ${\epsilon}_{ij}^{2}$ of the Kelvin creep element considering thermal damage, it can be expressed as:

21 $$\begin{array}{c}{\epsilon}_{ij}^{2}=\frac{{S}_{ij}^{2}}{2{G}_{2}\left(1-{D}_{T}\right)}\left[1-{exp}\left(-\frac{{G}_{2}\left(1-{D}_{T}\right)}{{\eta}_{1}\left(1-{D}_{T}\right)}t\right)\right]\end{array}$$

where ${G}_{2}$ is the shear modulus and ${\eta}_{1}$ is the viscosity coefficient of the viscoelastic element, both accounting for thermal damage effects.

For the strain ${\epsilon}_{ij}^{3}$ of the viscoplastic element considering thermo–stress coupled damage, irreversible plastic deformation has already occurred. Therefore, it is necessary to consider the rock’s yield function *F* and plastic potential function *Q*. Based on the associated flow rule (*F = Q*), the three-dimensional constitutive relationship of the viscoplastic element is given by^42^:

22 $$\begin{array}{c}{\epsilon}_{ij}^{3}=\frac{F}{2{\eta}_{2}\left(1-{D}_{TS}\right)}\frac{\partial F}{\partial{\sigma}_{ij}}\end{array}$$

Under normal and low-to-moderate temperature conditions, it is generally accepted that hydrostatic pressure (spherical stress tensor) has a negligible effect on creep, with deviatoric stress playing the dominant role. However, at elevated temperatures, the influence of hydrostatic pressure can no longer be ignored. The Drucker–Prager (*D–P*) criterion accounts for both the effect of the intermediate principal stress and hydrostatic pressure, thereby overcoming the limitations of the Mohr–Coulomb criterion, which neglects the intermediate principal stress, and the Von Mises criterion, which disregards the influence of hydrostatic stress on rock strength. As a result, compared to the Mohr–Coulomb and Von Mises criteria, the *D–P* criterion is more suitable for geomaterials, offers broader applicability, and has been widely adopted in numerical simulations. In fact, the influence of temperature and stress on the internal friction angle of rock is non-negligible^38,43^. Since variations in internal friction angle affect the plastic yield surface, a modified Drucker–Prager failure criterion considering thermo–stress damage is defined as ${F}_{{D}_{TS}}$:

23 $$\begin{array}{c}{F}_{{D}_{TS}}={\alpha}_{{D}_{TS}}{I}_{1}+\sqrt{{J}_{2}}-{k}_{{D}_{TS}}\end{array}$$

where ${I}_{1}$ and ${J}_{2}$ are the first stress invariant and the second deviatoric stress invariant, respectively. It should be emphasized that temperature effects are not introduced as an explicit additive term in the yield function. Instead, temperature dependence is embedded through the evolution of strength parameters. Specifically, cohesion and internal friction angle are formulated as functions of thermo–mechanical damage variables, i.e., $c=c(T,D)$and $\phi=\phi(T,D)$. As a result, the yield surface evolves continuously with temperature and damage accumulation.

${\alpha}_{{D}_{TS}}$ and ${k}_{{D}_{TS}}$ are defined as follows:

24 $$\begin{array}{c}\left\{\begin{array}{c}{\alpha}_{{D}_{TS}}=\frac{{sin}{\phi}_{0}\left(1-{D}_{TS}\right)}{\sqrt{9+3{{sin}}^{2}{\phi}_{0}\left(1-{D}_{TS}\right)}}\\{k}_{{D}_{TS}}=\frac{\sqrt{3}{c}_{0}\left(1-{D}_{TS}\right){cos}{\phi}_{0}\left(1-{D}_{TS}\right)}{\sqrt{3+{{sin}}^{2}{\phi}_{0}\left(1-{D}_{TS}\right)}}\end{array}\right.\end{array}$$

where ${\phi}_{0}$ is the internal friction angle of the rock at room temperature, and ${c}_{0}$ is the cohesion at room temperature. Through this formulation, the cohesion and internal friction angle degrade continuously with the coupled damage variable, resulting in dynamic evolution of the yield surface under thermo–mechanical coupling.

For conventional triaxial compression creep tests on rocks, the following relationship holds:

25 $$\begin{array}{c}\left\{\begin{array}{c}{\sigma}_{1}>{\sigma}_{2}={\sigma}_{3}\\{I}_{1}={\sigma}_{1}+{\sigma}_{2}+{\sigma}_{3}={\sigma}_{1}+2{\sigma}_{3}\\\sqrt{{J}_{2}}=\sqrt{\frac{{\left({\sigma}_{1}-{\sigma}_{2}\right)}^{2}+{\left({\sigma}_{2}-{\sigma}_{3}\right)}^{2}+{\left({\sigma}_{3}-{\sigma}_{1}\right)}^{2}}{6}}=\frac{{\sigma}_{1}-{\sigma}_{3}}{\sqrt{3}}\\{\sigma}_{m}=\frac{{\sigma}_{1}+{\sigma}_{2}+{\sigma}_{3}}{3}=\frac{{\sigma}_{1}+2{\sigma}_{3}}{3}\\{S}_{11}={\sigma}_{1}-{\sigma}_{m}=\frac{2\left({\sigma}_{1}-{\sigma}_{3}\right)}{3}\\F={\alpha}_{{D}_{TS}}\left({\sigma}_{1}+2{\sigma}_{3}\right)+\frac{{\sigma}_{1}-{\sigma}_{3}}{\sqrt{3}}+{k}_{{D}_{TS}}\\\frac{\partial F}{\partial{\sigma}_{1}}=\frac{\sqrt{3}+3{\alpha}_{{D}_{TS}}}{3}\end{array}\right.\end{array}$$

It should be noted that although Eq. (25) does not explicitly contain a temperature term, its temperature dependence arises through the strength parameters $c(T,D)$and $\phi(T,D)$. Accordingly, the yield function may be expressed as $F(\boldsymbol{\sigma};{\hspace{0.17em}}c(T,D),\phi(T,D\left)\right)=0$, indicating that temperature and damage effects are incorporated implicitly via parameter evolution, leading to continuous degradation of the yield surface under thermo–mechanical coupling.

By combining Eqs. (19) through (25), the creep equation of the TSD model for rock under three-dimensional stress conditions can be obtained as:

26 $$\begin{array}{c}{\epsilon}_{11}\left(t\right)=\left\{\begin{array}{c}\frac{{\sigma}_{1}-{\sigma}_{3}}{3{G}_{1}\left(1-{D}_{T}\right)}+\frac{{\sigma}_{1}+2{\sigma}_{3}}{9K\left(1-{D}_{T}\right)}+\frac{{\sigma}_{1}-{\sigma}_{3}}{3{G}_{2}\left(1-{D}_{T}\right)}\left[1-{exp}\left(-\frac{{G}_{2}\left(1-{D}_{T}\right)}{{\eta}_{1}\left(1-{D}_{T}\right)}t\right)\right]\text{}\text{}\text{}{\sigma}_{1}-{\sigma}_{3}<{\sigma}_{S}\\\frac{{\sigma}_{1}-{\sigma}_{3}}{3{G}_{1}\left(1-{D}_{T}\right)}+\frac{{\sigma}_{1}+2{\sigma}_{3}}{9K\left(1-{D}_{T}\right)}+\frac{{\sigma}_{1}-{\sigma}_{3}}{3{G}_{2}\left(1-{D}_{T}\right)}\left[1-{exp}\left(-\frac{{G}_{2}\left(1-{D}_{T}\right)}{{\eta}_{1}\left(1-{D}_{T}\right)}t\right)\right]\\+\frac{{F}_{{D}_{TS}}\left(\sqrt{3}+3{\alpha}_{{D}_{TS}}\right)}{6{\eta}_{2}\left(1-{D}_{TS}\right)}t\text{}\text{}\text{}\text{}\text{}\text{}\text{}\text{}\text{}\text{}\text{}\text{}\text{}\text{}\text{}\text{}\text{}\text{}\text{}\text{}\text{}\text{}\text{}\text{}\text{}\text{}\text{}\text{}\text{}\text{}\text{}\text{}\text{}\text{}\text{}\text{}\text{}\text{}\text{}\text{}\text{}\text{}\text{}\text{}\text{}\text{}\text{}\text{}\text{}\text{}\text{}\text{}\text{}\text{}\text{}\text{}\text{}\text{}\text{}\text{}\text{}\text{}\text{}\text{}\text{}\text{}\text{}\text{}\text{}\text{}\text{}\text{}\text{}\text{}\text{}\text{}\text{}\text{}\text{}\text{}\text{}\text{}\text{}\text{}\text{}\text{}\text{}\text{}\text{}\text{}\text{}\text{}\text{}\text{}\text{}\text{}\text{}\text{}{\sigma}_{1}-{\sigma}_{3}>{\sigma}_{S}\end{array}\right.\end{array}$$

Through this formulation, thermo–mechanical coupling is reflected by the dynamic evolution of strength parameters and the associated continuous degradation of the yield surface, rather than by a fixed yield envelope with constant material properties.

## Validation of the thermo–stress coupled creep damage constitutive model

In this section, the TSD creep constitutive model is validated by calibrating model parameters using granite creep test data from the literature^43^. A comparative analysis between theoretical predictions and experimental results is conducted to assess the accuracy and applicability of the TSD model. In this validation, the creep strain $\epsilon$, stress $\sigma$, and time $t$ are obtained from experimental data, while the model parameters to be identified in the three-dimensional formulation include: ${G}_{1}$, ${G}_{2}$, $K$, ${\eta}_{1}$, ${\eta}_{2}$, $E$, $\alpha$, $a$, $b$, $c$, and $q$. Given the large number of parameters and the model’s inherent complexity, traditional least-squares methods exhibit limited optimization capability in the parameter inversion process. Therefore, a two-stage fitting strategy combining global optimization and local nonlinear least-squares fitting is adopted to ensure high-quality parameter estimation in a complex parameter space.

### Granite creep test

**Fig. 7:**
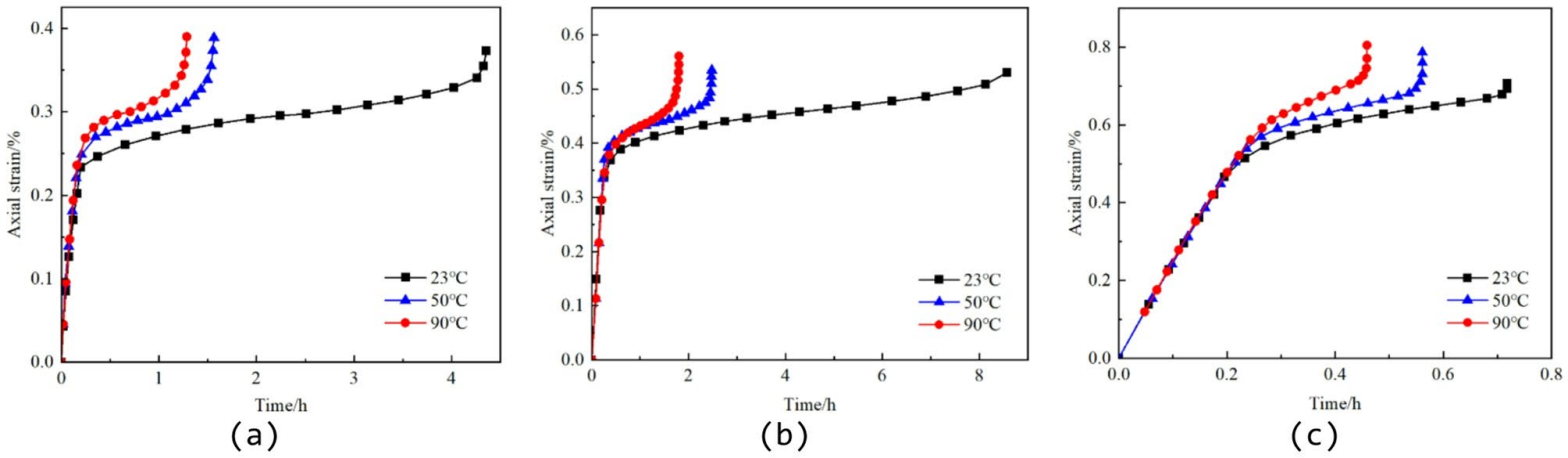
Creep curves of granite specimens under different confining pressures and temperature conditions. (**a**) 2 MPa confining pressure, (**b**) 10 MPa confining pressure, (**c**) 30 MPa confining pressure.

Reference^43^ conducted conventional triaxial creep experiments on granite specimens under three temperature levels (23 °C, 50 °C, and 90 °C), as shown in Fig. 7. The tested material was granite obtained from a depth of 450–550 m in the Beishan area of Gansu Province, China, characterized as medium- to fine-grained granodiorite. Cylindrical specimens with dimensions of 50 mm in diameter and 100 mm in height were prepared for testing. The experiments were carried out using an MTS815 rock mechanics testing system, which provides a maximum axial load of 4600 kN, a maximum confining pressure of 140 MPa, and a temperature control range from room temperature to 200 °C. The loading procedure consisted of two stages: an initial loading stage and a constant-load creep stage. After the target temperature and confining pressure were reached, the test entered the initial loading stage, during which the axial stress was increased at a loading rate of 0.3 MPa/s until the predetermined stress level was achieved; subsequently, the test proceeded to the constant-load stage, where the axial load was maintained until specimen failure. The applied stress levels, confining pressures, and temperature conditions for each specimen are summarized in Table 1.

**Table 1:** Temperature–confining pressure–stress loading conditions.

Specimen	Temperature/℃	Confining pressure/MPa	Differential stress/MPa
1	23	2	157.8
2	50	2	165.3
3	90	2	178.8
4	23	10	227.8
5	50	10	239.48
6	90	10	224.9
7	23	30	365.85
8	50	30	327.9
9	90	30	374.8

According to Ref.^43^, the axial stress levels under each temperature and confining pressure condition were selected based on a proportional stress criterion relative to the corresponding short-term peak strength. The applied stress was set below the peak strength to ensure sustained creep deformation under constant load, while allowing the tertiary creep stage and eventual failure to occur within the experimental duration. Therefore, the selected stress levels represent stress ratios under given thermo–mechanical conditions rather than independently determined long-term strength values. It should be noted that the creep duration in Ref.^43^ was on the order of several hours, which is typical for laboratory-based high-temperature triaxial creep tests due to practical time and equipment constraints. In this study, the term “long-term” refers to the constitutive representation of time-dependent creep mechanisms and the ability to reproduce the complete three-stage creep evolution (primary–secondary–tertiary), rather than field-scale service life over years. Extrapolation to true long-term engineering time scales requires dedicated long-duration experiments, which will be addressed in future research.

The experimental results indicate that granite exhibits three typical creep stages under different temperature conditions: the primary (transient) creep stage, the steady-state creep stage, and the tertiary (accelerating) creep stage. Under the same applied constant stress, the instantaneous strain of granite increases with temperature, suggesting a reduction in instantaneous elastic modulus as temperature rises. Moreover, as the temperature increases, the duration of the steady-state creep stage shortens and the steady-state creep rate increases significantly. These observations confirm that elevated temperatures accelerate the creep damage process in granite and provide essential support for fitting the parameters of the high-temperature creep constitutive model.

### Parameter identification and model validation

For the fitting of parameters ${G}_{1}$, ${G}_{2}$, $K$, ${\eta}_{1}$, ${\eta}_{2}$, $E$, $\alpha$, $a$, $b$, $c$, and $q$ in the second equation of the granite TSD creep constitutive model, a two-stage fitting strategy combining global optimization and local nonlinear least-squares fitting is employed to ensure high-quality results in a complex parameter space. First, the Differential Evolution (DE) algorithm is applied to perform a global search for multiple unknown positive parameters of the model. DE is a stochastic optimization method based on the concept of population evolution, capable of executing global optimization for nonlinear and multi-modal objective functions without requiring gradient information. The Differential Evolution (DE) algorithm was implemented with the following parameter settings. The population size was set to 50, which provides a balance between global search capability and computational efficiency. The mutation factor was taken as 0.8, and the crossover probability was set to 0.9 to enhance exploration ability in the early stage of optimization. The maximum number of iterations was specified as 500. The convergence criterion was defined such that the optimization process was terminated when the variation of the objective function value between successive generations was less than 1 × 10⁻⁶. These settings were adopted to ensure stable convergence and sufficient global search performance in the multi-parameter identification process.The objective function is defined as follows^44,45^:

27 $$\begin{array}{c}\underset{\theta}{min}\sum\limits_{i=1}^{n{\sum}^{2}}\left[{\varepsilon}_{model}\left({t}_{i};\theta\right)-{\varepsilon\left({t}_{i}\right)}_{exp}\left[\right]\right]\end{array}$$

where $\theta$ is the vector of parameters to be optimized, ${\varepsilon}_{model}$ is the strain predicted by the model, and ${\varepsilon}_{exp}$ is the strain measured in the experiment.

The objective of this stage is to obtain a set of physically reasonable initial parameters that provide an effective starting point for subsequent local refinement. On this basis, the nonlinear least-squares method is employed to perform fine fitting of the model^46^.

**Fig. 8:**
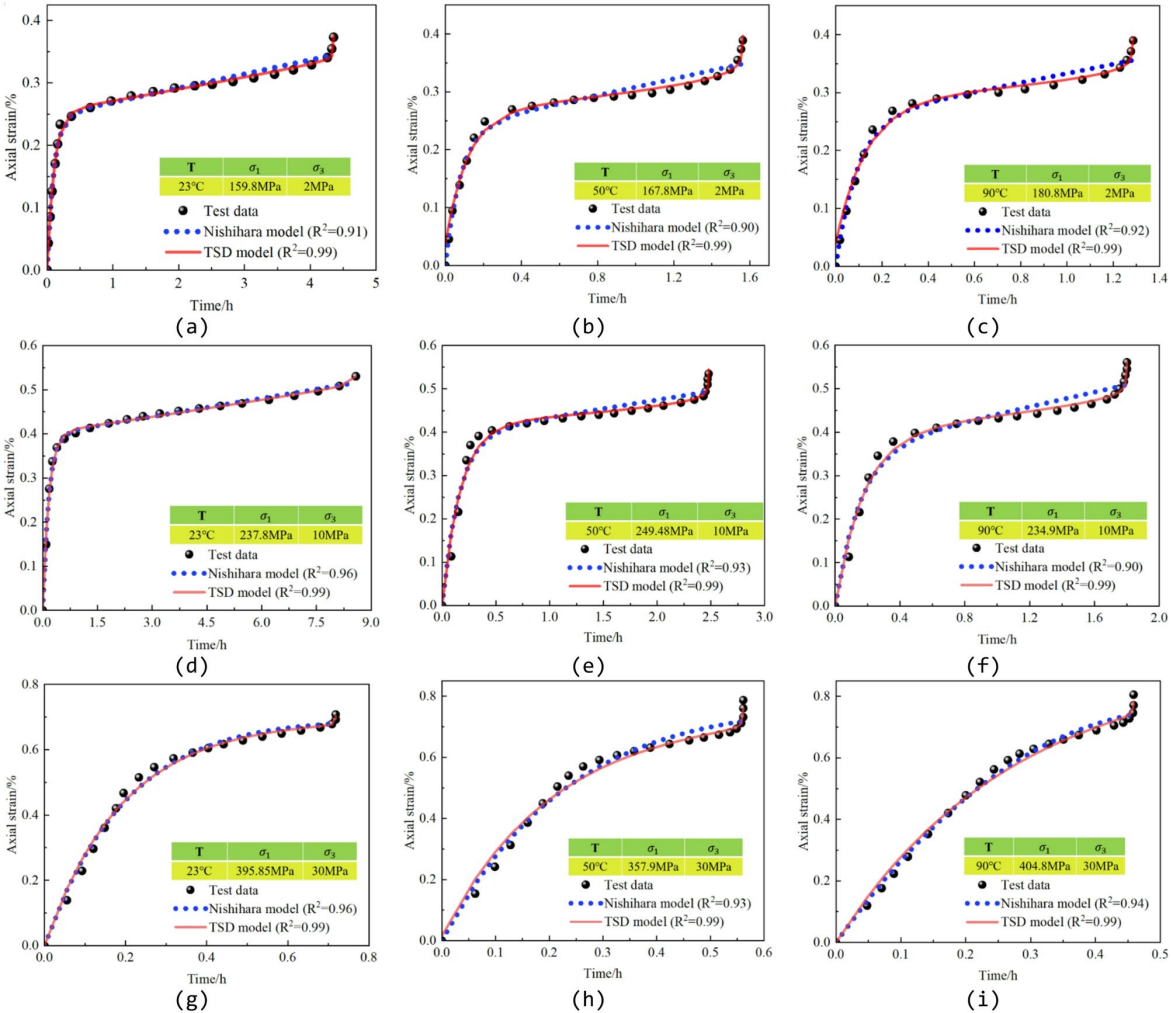
Fitted curves of the TSD creep model. (**a**) Specimen 1, (**b**) Specimen 2, (**c**) Specimen 3, (**d**) Specimen 4, (**e**) Specimen 5, (**f**) Specimen 6, (**g**) Specimen 7, (**h**) Specimen 8, (**i**) Specimen 9.

The parameter identification results are summarized in Table 2, and the experimental data together with the corresponding model fitting curves are presented in Fig. 8. Comparative analysis indicates that the deviation between the experimental data and the fitted curves of the proposed TSD creep constitutive model is relatively small, with coefficients of determination (R²) exceeding 0.99 in all cases. These results demonstrate the high fitting accuracy and reliability of the developed model. The proposed TSD creep constitutive model is capable of accurately characterizing the high-temperature creep behavior of granite and reproducing the complete three-stage creep process, including instantaneous creep, steady-state creep, and accelerated creep. In particular, the model shows strong capability in describing the nonlinear strain acceleration during the tertiary creep stage.

**Table 2:** Parameter fitting results.

Specimen	${G}_{1}$/GPa	${G}_{2}$/MPa	K/GPa	${\eta}_{1}$/MPa.h	${\eta}_{2}$/MPa.h	E/GPa	α	a	b	c	q	${t}_{F}$/h	*R* ^2^
1	28	208.95	47.33	22.51	1576.81	/	/	/	/	/	17.27	4.34	0.99
2	28	515.90	47.33	48.58	2258.34	71	0.03	5.98	9.16	-4.78	5.98	1.56	0.99
3	28	734.74	47.33	80.15	3799.14	71	0.01	9.63	3.05	3.89	5.28	1.29	0.99
4	28	185.17	47.33	33.07	4256.53	/	/	/	/	/	10	8.58	0.99
5	28	381.70	47.33	65.88	3170.40	71	0.23	1.69	2.19	3.5	6.01	2.47	0.99
6	28	549.70	47.33	104.74	5247.94	71	0.01	-1.06	-3.02	-4.62	5.25	1.81	0.99
7	28	180.72	47.33	34.47	5019.42	/	/	/	/	/	3.09	0.72	0.99
8	28	245.83	47.33	49.13	4167	71	0.25	0.14	0.26	1.2	2.96	0.56	0.99
9	28	354.44	47.33	99.48	7562	71	0.39	0.51	0.32	1.41	2.86	0.46	0.99

To further evaluate the applicability and comparative performance of the model, the classical Nishihara model was introduced for fitting comparison. The results show that the average coefficient of determination for the Nishihara model is approximately 0.92, which is significantly lower than that of the TSD model. Notably, during the accelerated creep stage, the classical Nishihara model fails to effectively capture the rapid strain growth, resulting in relatively large fitting deviations. In contrast, the TSD model exhibits higher fitting accuracy and stronger adaptability across the entire creep process, highlighting its advantages in representing high-temperature creep behavior.

In the present study, the instantaneous elastic modulus is determined from the initial linear portion of the experimental stress–strain curves and is taken as 71 GPa for model calibration. Although numerous studies have reported that elastic modulus may vary with temperature and loading conditions, the variation within the temperature range considered (23–90 °C) is relatively limited compared with the pronounced evolution observed during creep damage development. Therefore, the elastic modulus is treated as constant to focus on the time-dependent deformation and strength degradation mechanisms. Incorporating temperature-dependent elastic parameters would increase parameter coupling and model complexity, which may be addressed in future extensions of the model.

## Creep damage analysis

**Fig. 9:**
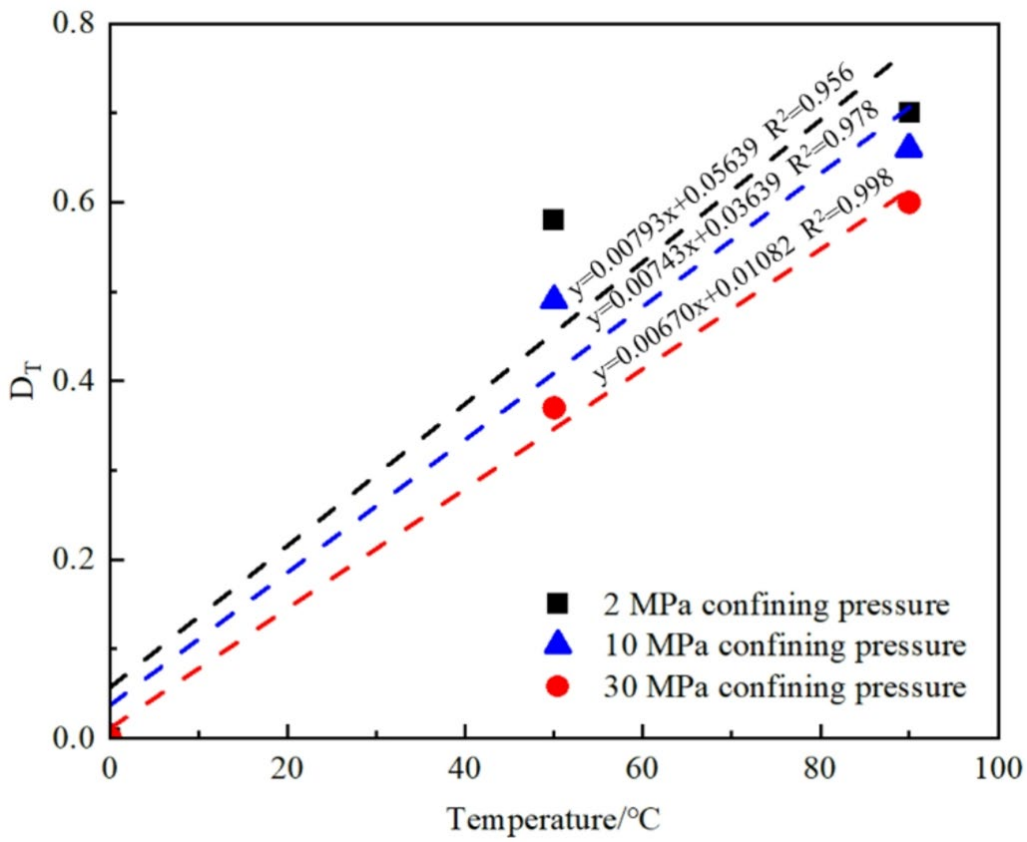
Evolution curve of the thermal damage factor D_T_.

**Fig. 10:**
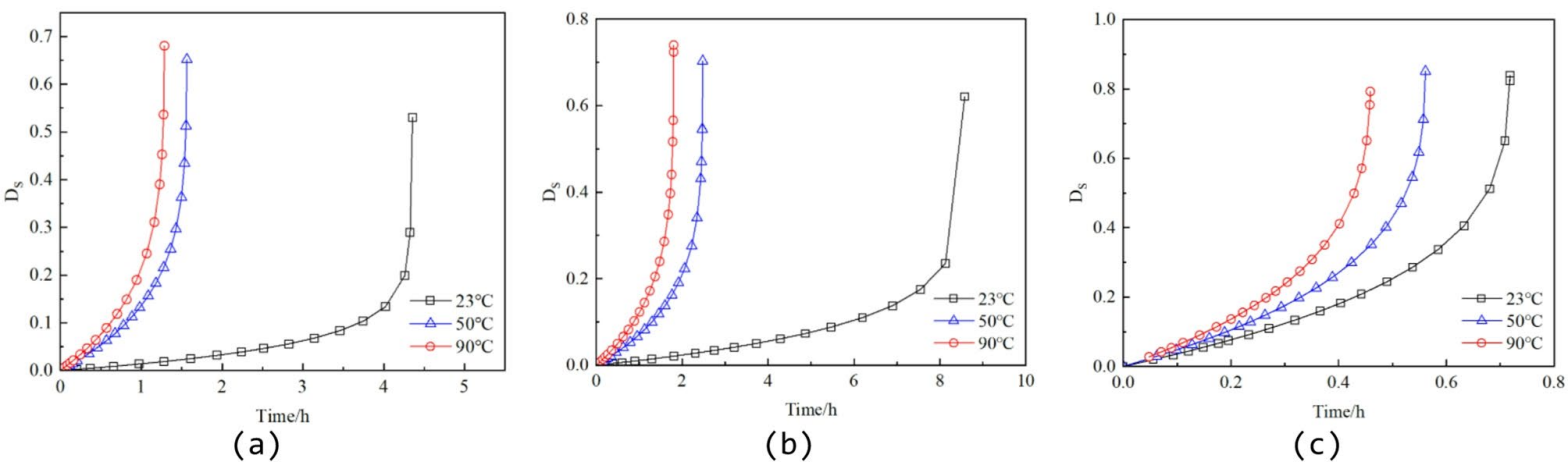
Evolution curve of the stress damage factor D_S_. (**a**) 2 MPa confining pressure, (**b**) 10 MPa confining pressure, (**c**) 30 MPa confining pressure.

**Fig. 11:**
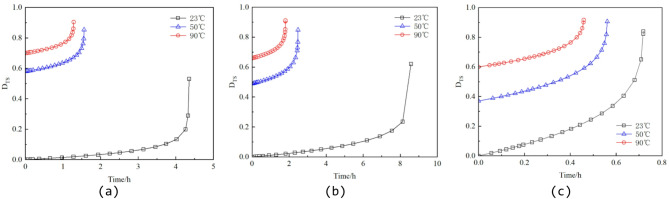
Evolution curve of the thermo–stress coupled damage factor D_TS_. (**a**) 2 MPa confining pressure, (**b**) 10 MPa confining pressure, (**c**) 30 MPa confining pressure.

By substituting the data from Table 2 into Eqs. (6), (12), and (14), the stress damage factors for different specimens can be calculated. Figures 9, 10 and 11 present the variations of the thermal damage factor, stress damage factor, and thermo–stress coupled damage factor with time for the different specimens.

From Fig. 9, it can be observed that the thermal damage factor D_T_ remains constant throughout the entire loading process, indicating that it does not evolve with time and is primarily determined by the applied temperature. Under the same temperature conditions, an increase in confining pressure significantly suppresses the initiation and propagation of microcracks, thereby reducing the damage rate. For example, at 50 °C, the thermal damage factor D_T_ is 0.57, 0.48, and 0.37 under confining pressures of 2 MPa, 10 MPa, and 30 MPa, respectively. The increase in confining pressure reduces intermolecular spacing, leading to a denser structure and consequently diminishing the effect of thermal damage, although the overall impact remains relatively minor.

From Figs. 10 and 11, it is evident that both the stress damage factor D_S_ and the thermo–stress coupled damage factor D_TS_ evolve slowly during the initial stage, followed by a sharp increase in the later stage, reflecting the lag effect in the accumulation of stress damage. Under the same confining pressure, higher temperatures lead to faster growth of the damage factor curves and higher final peak values. This behavior can be attributed to the intensified lattice thermal vibration at elevated temperatures, which exacerbates stress concentration at crack tips, accelerates crack propagation, and thus promotes faster damage evolution.

**Fig. 12:**
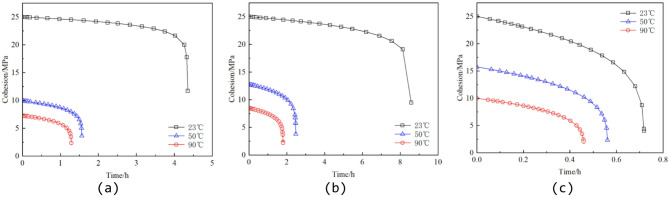
Evolution curve of cohesion. (**a**) 2 MPa confining pressure, (**b**) 10 MPa confining pressure, (**c**) 30 MPa confining pressure.

**Fig. 13:**
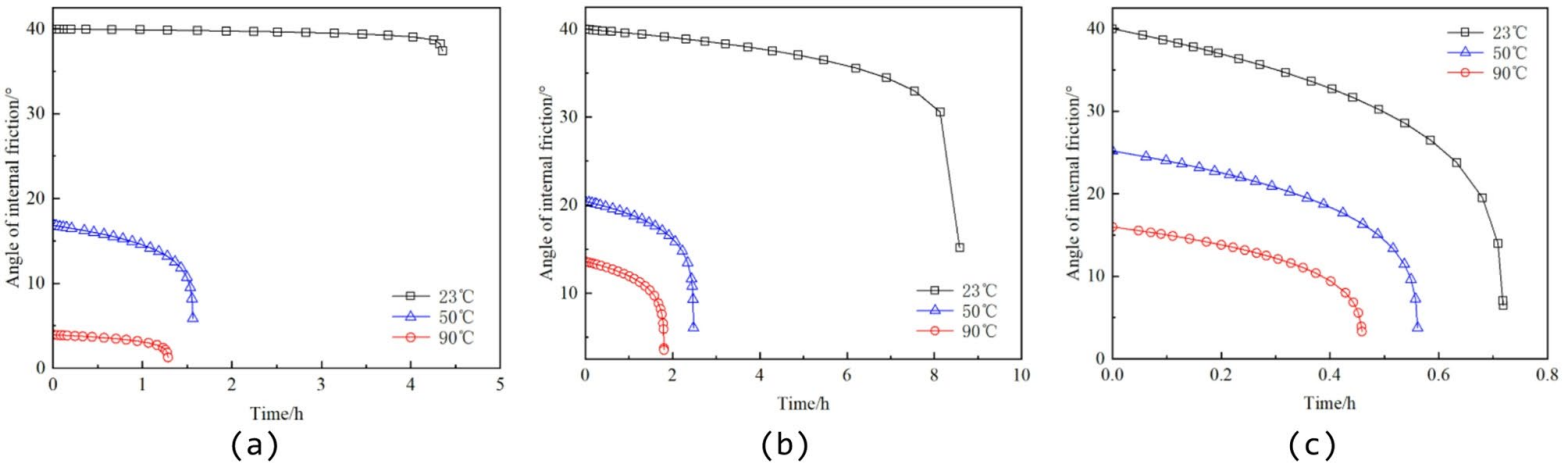
Evolution curve of internal friction angle. (**a**) 2 MPa confining pressure, (**b**) 10 MPa confining pressure, (**c**) 30 MPa confining pressure.

To analyze the influence of thermo–stress coupled damage on rock parameters, cohesion and internal friction angle are selected as examples. The values of D_T_, D_S_, and D_TS_ are substituted into Eq. (22), and the corresponding evolution curves are plotted, as shown in Figs. 12 and 13.

From Figs. 12 and 13, it can be observed that both cohesion and internal friction angle of the rock specimens exhibit a pronounced degradation trend with increasing time and temperature. The results indicate that as temperature rises, the initial values of cohesion and internal friction angle decrease significantly, and their rates of decay increase markedly. For example, under a confining pressure of 2 MPa at 23 °C, cohesion decreases gradually from an initial value of 25 MPa to approximately 11.74 MPa, while the internal friction angle drops from 40° to 37.41°, indicating only minor overall degradation. In contrast, at 50 °C, cohesion rapidly declines from 9.93 MPa to 3.64 MPa, and the internal friction angle decreases from 16.83° to 5.87°, demonstrating a clear high-temperature softening trend. At 90 °C, the mechanical properties of the rock deteriorate further, with cohesion reduced to 2.39 MPa and the internal friction angle falling below 1.3°, indicating an almost complete loss of frictional shear resistance.

Overall, the pronounced effect of temperature on mechanical parameters primarily originates from the enhanced thermal disturbance to the rock microstructure at elevated temperatures^47,48^. High temperatures intensify the thermal vibration of mineral lattices, weakening the cementation between grains and leading to a rapid reduction in cohesion. Simultaneously, elevated temperatures promote the initiation and propagation of microcracks, reducing intergranular frictional contact and facilitating slip, which in turn causes a continuous decrease in the internal friction angle. Moreover, high temperatures may trigger a brittle-to-plastic transition in the rock, shifting the dominant failure mechanism from cementation-controlled brittle fracture to slip- and softening-dominated plastic failure. In summary, temperature not only lowers the overall strength of the rock but also accelerates the degradation of its strength parameters, thereby intensifying the evolution of structural damage under thermo–stress coupling.

It should be clarified that the evolution of cohesion and internal friction angle shown in Fig. 12 is derived from the proposed constitutive model through parameter inversion and damage evolution relationships. These results represent model-predicted equivalent strength parameter evolution, rather than independently measured experimental data. Their purpose is to analyze the trend of strength degradation under thermo–mechanical coupling and to interpret the mechanical implications of the coupled damage framework. From a qualitative perspective, the observed reduction in cohesion with increasing temperature is consistent with previous studies reporting thermal weakening of granite due to thermally induced microcrack initiation, propagation, and damage accumulation^49^. However, quantitative differences in degradation magnitude compared with some reported results may arise from variations in lithology and microstructure, thermal loading paths (real-time heating versus thermal pretreatment), confining pressure and stress levels, as well as differences in parameter identification procedures and adopted failure criteria. Moreover, prior investigations have indicated that thermal crack initiation in granite may occur within a moderate temperature range (approximately 70–100 °C), which can significantly accelerate cohesion degradation^50^. Therefore, the relatively pronounced reduction observed in Fig. 12 may be interpreted as the combined effect of thermo–mechanical damage evolution under the specific loading and modeling framework adopted in this study.

## Discussion

1. The thermo–stress coupled creep damage model developed in this study can be effectively applied to analyze the creep behavior of rocks in high-temperature environments and is suitable for scenarios such as deep geological repositories, mining engineering, and geothermal development. However, for rock engineering in cold regions^51^, the model has not yet demonstrated sufficient applicability. Future research will therefore focus on developing a creep damage model applicable over a broader temperature range to enhance its practicality and universality. In addition, the proposed TSD model has been experimentally validated only under three temperature conditions—23 °C, 50 °C, and 90 °C—while rock temperatures in actual engineering applications are often higher, which to some extent limits the model’s applicability and persuasiveness.
2. The damage factors introduced in this study comprehensively account for the coupling effects of temperature and stress. In subsequent work, additional influencing factors—such as seepage effects, chemical reaction processes, and the heterogeneity of rock materials—will be incorporated to improve the model’s adaptability to complex geological environments and its engineering applicability.
3. For parameter identification, a hybrid fitting strategy combining global optimization and local least-squares methods is adopted in this study. Compared with the traditional least-squares approach, this method demonstrates superior performance in both fitting accuracy and efficiency. As creep models continue to evolve and increase in complexity, the number of parameters will also grow, placing higher demands on identification algorithms^52^. Therefore, future work should introduce more efficient and robust optimization algorithms to meet the parameter identification requirements of complex models.
4. In the present study, validation of the TSD model is conducted using published high-temperature triaxial creep data for parameter calibration and comparative analysis. This approach enables assessment of the model’s capability to reproduce the complete three-stage creep behavior and to ensure theoretical consistency. However, the validation remains within the scope of existing constant-stress datasets and does not include prospective verification based on independently conducted experiments. Moreover, due to the limited availability of high-temperature creep data under complex loading paths, validation under step-loading or variable-stress conditions has not been performed. Although the three-dimensional constitutive formulation is not restricted to specific stress levels, further verification under more diverse stress paths will be pursued when corresponding experimental data become available.

It should also be noted that the damage variables introduced in this study are internal state variables within a continuum mechanics framework. They represent equivalent macroscopic damage evolution reflected in mechanical response and are not directly equivalent to observable crack density or microcrack population. Therefore, their physical interpretation requires indirect validation through multi-scale experimental techniques. Future research will incorporate approaches such as computed tomography (CT), scanning electron microscopy (SEM), and acoustic emission (AE) monitoring to establish quantitative linkage between model parameters and microstructural evolution, thereby enhancing the physical credibility and predictive robustness of the model.

(5) Long-term strength is an important engineering indicator in rock creep analysis. Within the proposed framework, long-term strength under a given temperature and confining pressure can be conceptually defined as the critical stress level below which the damage evolution does not trigger tertiary creep instability, i.e., a stable creep stage can be maintained without accelerated strain growth. Since the yield surface in the present model evolves with the coupled damage variable through $c(T,D)$and $\phi(T,D)$, the corresponding long-term strength can be evaluated by combining the damage-dependent strength parameters with the yield criterion. A dedicated quantitative determination of long-term strength and its experimental verification will be pursued in future work.

## Conclusions

Building upon classical creep models, this study proposes a novel thermo–stress coupled creep damage constitutive model—the TSD creep model—and applies it to parameter identification and validation using granite creep test curves under various temperature and confining pressure conditions. The main conclusions are as follows:

1. To account for the effects of temperature and stress on microcrack propagation during granite creep, a thermal damage factor D_T_, a stress damage factor D_S_, and a thermo–stress coupled damage factor D_TS_ are introduced. On this basis, elastic and viscous elements incorporating damage effects are developed to replace the elastic and viscous elements in the classical Nishihara model, thereby establishing the TSD creep model. The one-dimensional creep constitutive equation of the model is derived accordingly.
2. Based on the Drucker–Prager yield criterion, the factor D_TS_ is incorporated to account for thermo–stress coupled damage, and the relationships between the damage factor and rock strength parameters—such as cohesion and internal friction angle—are established. Subsequently, the three-dimensional creep constitutive equation of the TSD model and its specific form under conventional triaxial compression test conditions are derived.
3. A hybrid fitting strategy combining global optimization and local nonlinear least-squares methods is employed to perform inversion analysis of granite creep test curves, obtaining the parameters for each model component. Substituting the fitted parameters into the TSD constitutive equation and comparing with experimental data shows that the model can accurately capture the creep behavior of granite under different temperature and stress conditions, exhibiting particularly good agreement during the nonlinear accelerating creep stage.
4. The evolution characteristics of the damage factors are analyzed in conjunction with micro-mechanical mechanisms. The results indicate that the thermal damage factor D_T_ remains constant during loading, is primarily controlled by the applied temperature, and does not vary with time. An increase in confining pressure can suppress microcrack propagation to some extent, reducing the degree of thermal damage, although the effect is relatively minor. Both the stress damage factor D_S_ and the coupled damage factor D_TS_ show delayed growth with loading time, followed by a rapid increase in the later stage, reflecting a pronounced accumulation of damage. Under high-temperature conditions, crack propagation is markedly accelerated, intensifying damage evolution. Moreover, thermo–stress coupled damage significantly reduces the cohesion and internal friction angle of the rock, producing a pronounced high-temperature softening effect that leads to a substantial decrease in shear strength; in extreme cases, the rock mass may even lose its frictional shear resistance entirely.

## Data Availability

Data will be made available on request.
